# Disruption of Tfh:B Cell Interactions Prevents Antibody-Mediated Rejection in a Kidney Transplant Model in Rats: Impact of Calcineurin Inhibitor Dose

**DOI:** 10.3389/fimmu.2021.657894

**Published:** 2021-05-31

**Authors:** Louisa Steines, Helen Poth, Antonia Schuster, Kerstin Amann, Bernhard Banas, Tobias Bergler

**Affiliations:** ^1^ Department of Nephrology, University Hospital Regensburg, Regensburg, Germany; ^2^ Department of Nephropathology, University Hospital Erlangen, Erlangen, Germany

**Keywords:** antibody-mediated rejection, donor-specific antibodies, kidney transplantation, T follicular helper cells, B cell activation, calcineurin inhibitor

## Abstract

We aimed to investigate the mechanisms of humoral immune activation in ABMR using a MHC-mismatched rat kidney transplant model. We applied low dose cyclosporine A (loCNI) to allow donor-specific antibody (DSA) formation and rejection and high dose cyclosporine A (hiCNI) for non-rejection. DSA and leukocyte subsets were measured by flow cytometry. Germinal centers (GC), T follicular helper cells (Tfh), plasma cells and interleukin-21 (IL-21) expression were analyzed by immunofluorescence microscopy. Expression of important costimulatory molecules and cytokines was measured by qRT-PCR. Allograft rejection was evaluated by a nephropathologist. We found that DSA formation correlated with GC frequency and expansion, and that GC size was linked to the number of activated Tfh. In hiCNI, GC and activated Tfh were virtually absent, resulting in fewer plasma cells and no DSA or ABMR. Expression of B cell activating T cell cytokine IL-21 was substantially inhibited in hiCNI, but not in loCNI. In addition, hiCNI showed lower expression of ICOS ligand and IL-6, which stimulate Tfh differentiation and maintenance. Overall, Tfh:B cell crosstalk was controlled only by hiCNI treatment, preventing the development of DSA and ABMR. Additional strategies targeting Tfh:B cell interactions are needed for preventing alloantibody formation and ABMR.

## Introduction

Antibody-mediated rejection (ABMR) is a major cause of allograft failure in kidney transplantation (Ktx) (1). Donor-specific antibodies (DSA) are responsible for initiating ABMR and their serological presence, whether pre-existing or formed after transplantation (“*de novo*”), is associated with poorer graft survival (2–6). We aimed to examine the mechanisms of humoral immune activation in a clinically relevant model of chronic kidney allograft rejection in order to identify novel strategies for immunosuppressive intervention.

High affinity antibodies arise from the germinal center (GC) reaction. GC are transient structures that form in the follicles of secondary lymphoid organs (SLO). Here, antigen-specific B cells undergo somatic hypermutation (SHM) of their immunoglobulin (Ig) genes and perform class switch recombination (CSR) to generate affinity-matured antibodies with specific effector functions (7). As a result, highly specific long-lived plasma cells and memory B cells are generated (8). Beyond this, certain clinical observations suggest that processes integral to the GC reaction control the development of ABMR. For instance, the mean fluorescence intensity (MFI) of DSA, which reflects alloantibody affinity and concentration, affects ABMR risk in Ktx patients (9–12). Furthermore, the IgG subclass of DSA has been linked to the phenotype and clinical course of ABMR (13). Thus, the affinity maturation and Ig (sub-) class switch of DSA, which are regulated in the GC reaction, impact the development and course of ABMR.

T follicular helper cells (Tfh) are a specialized T helper cell subset with the primary function of activating cognate B cells, as reviewed by Vinuesa *et al. (* 14). The expression of the B cell chemokine receptor CXCR5 guides them to the B cell follicle, where they provide essential signals driving the GC reaction. Tfh expression of CD40 ligand and interleukin-21 (IL-21) stimulate B cell proliferation, SHM and CSR (15). Excessive Tfh activation occurs in autoimmune diseases (16). In Ktx, Tfh involvement has been implicated by reports linking circulating Tfh (cTfh) to pre-sensitization and rejection (17, 18). Moreover, a recent study showed IL-21, the canonical Tfh cytokine, is able to induce B cell differentiation and alloantibody formation in peripheral blood cells from Ktx patients (19). Other studies linked IL-21 expression to allograft rejection in Ktx patients (20, 21) and Htx patients (22). However, it is uncertain to what extent cTfh reflect ongoing processes in the SLO. A number of studies suggest that cTfh represent memory Tfh (23, 24), but some cTfh may represent pre-GC Tfh (25), raising questions about observations based on cTfh. Direct evidence of Tfh involvement in SLO in Ktx patients is lacking.

Since SLO tissue from Ktx patients is not easily accessible, animal models may provide important mechanistic insights. Previous models of ABMR in Ktx have been based on pre-sensitization and pre-formed alloantibodies (26, 27) and have thus been inappropriate to study the role of GC and Tfh in *de novo* allosensitization and ABMR. To study the role of GC and Tfh:B cell interactions in this setting, we used a unique model of ABMR in rats based on generation of *de novo* DSA due to under-immunosuppression. Using this model closely resembling clinical Ktx, we examined the generation of DSA, B cell activation in GC, the activation of Tfh in SLO, as well as molecules involved in Tfh:B cell crosstalk.

## Materials and Methods

### Rat Kidney Transplantation (Ktx) Model

Animal experiments were approved by local authorities (Regierung von Unterfranken) and performed according to animal protection laws. MHC-mismatched allogeneic Ktx was performed using Brown Norway rats (BN) as donors and Lewis rats (LEW) as recipients (Charles River Laboratories, Sulzfeld, Germany, 200–250 g), as previously described (28–30). In brief, BN kidneys were explanted, flushed with cold saline and transplanted orthotopically. Cold and warm ischemia times were approximately 35 and 30 min, respectively. Nephrectomy of the remaining recipient kidney was performed at the end of the surgery.

### Experimental Rat Treatments

**Figure 1:**
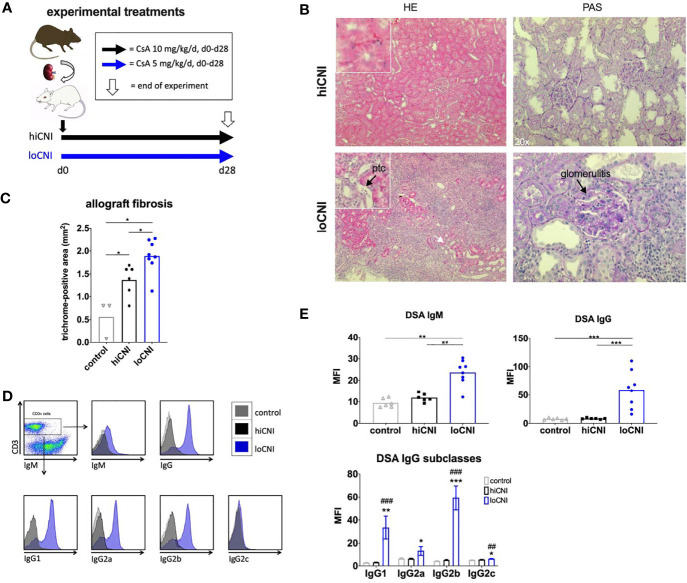
Experimental treatments, rejection, fibrosis and DSA formation after Ktx. **(A)** Ktx groups and CsA treatment, **(B)** allograft histology showing peritubular capillaritis (ptc, arrow) and glomerulitis (arrow) in HE and PAS staining. **(C)** allograft fibrosis assessed by trichrome staining in control BN kidneys (n=3), and hiCNI (n=6) and loCNI (n=8) allografts **(D)** Representative flow cytometry data of DSA IgM, IgG and IgG subclasses measured by flow crossmatch and **(E)** DSA shown as MFI values in serum from controls (n=6), hiCNI (n=6) and loCNI (n=8). Data is shown as mean and individual data points; statistical significance between groups is shown as *p ≤ 0.05, **p<0.01, ***p<0.001. For DSA IgG subclasses, data is shown as mean ± SEM; *p ≤ 0.05, **p<0.01, and ***p<0.001 for loCNI vs. hiCNI and ^##^p<0.01, and ^###^p<0.001 for loCNI vs. control.

To allow DSA formation and chronic rejection, rats were treated with low dose cyclosporine A (CsA 5 mg/kg/d, “loCNI”). The non-rejecting group received high dose CsA (10 mg/kg/d, “hiCNI”). CsA was administered orally by daily gavage for 28 days, beginning on the day of Ktx. CsA trough levels were measured by routine testing in the onsite clinical chemistry department by liquid chromatography coupled with tandem mass spectrometry. Untreated LEW rats served as controls. Rats were sacrificed 28 days after Ktx, when spleen and kidney allografts were removed and divided into sections for flow cytometry, fixed in paraffin, or snap-frozen in N_2_ and stored at -80 °C. **Figure 1A** shows the experimental treatments. **Table 1** shows the experimental groups.

**Table 1:** Experimental groups.

Group	Abbreviation	(n=)
Untreated Lewis rats	control	5-9
Ktx CsA 10mg/kg/d d28	hiCNI	6
Ktx CsA 5 mg/kg/d d28	loCNI	8

### Allograft Histology, Rejection and Fibrosis

Paraffin sections were prepared as previously described (31). After staining with hematoxylin and eosin (HE) and periodic acid schiff (PAS), the histomorphological alterations were classified according to the Banff classification 2017 (32) by an experienced nephropathologist in a blinded manner. C4d staining was performed using rabbit anti-rat C4d antibody (Hycultec HP8034, Beutelsbach, Germany) as previously described (28). Trichrome staining was performed as previously described (28).

### Donor-Specific Antibodies

DSA were measured by flow crossmatch as previously described (30). Briefly, donor BN splenocytes were incubated with heat-inactivated recipient serum for 30 minutes at 4°C and then washed. Cells were then stained using mouse anti-rat IgM-PE (ThermoFisher, 12-0990, Waltham, USA), chicken anti-rat IgG-Alexa Fluor647 antibody (ThermoFisher, A21472), anti-rat IgG1-APC (ThermoFisher 17-4812), mouse anti-rat IgG2a-PE Cy7 (ThermoFisher 25-4817), mouse anti-rat IgG2b-PE (ThemoFisher 12-4815), or mouse anti-rat IgG2c-biotin (BD 553909, Heidelberg, Germany) and streptavidin-BV421 (Biolegend 405226, San Diego, USA). Finally, cells were stained for CD3-FITC (ThermoFisher 11-0030) and measured by flow cytometry. Data is shown for CD3^+^-gated cells, to exclude unspecific binding of antibodies by Fc-receptors, and is restricted to DSA against MHC class I since MHC class II is not expressed on T cells.

### Flow Cytometry

Rat spleens were macerated and spleen cell suspensions separated by ficoll gradient centrifugation, as described before (30). Cells were blocked using 10% BSA PBS and stained with mouse anti-rat CD45R-PE-Cy7 (ThermoFisher 25-0460), mouse anti-rat CD11b/c-PE (ThermoFisher 12-0110-82), mouse anti-rat CD3-APC (ThermoFisher 17-0030-82) and hamster anti-mouse CD27-biotin (Serotec/Biorad MCA-4701B, Puchheim, Germany) and Streptavidin-APC (BD, 554067).

### Immunofluorescence Microscopy

3 μm formalin-fixed, paraffin-embedded sections were prepared and stained, as previously described (28). Cell nuclei were stained with DAPI (Hoechst 33342, Molecular Probes H-1399, Netherlands). Primary antibodies used were anti-rat IgG-Alexa Fluor 647 (ThermoFisher, A21472) for plasma cells, anti-IL-21 (Bioss bs-2621R-a350, Boston, USA), anti-CD20 (Santacruz sc-393894, Dallas, USA) for B cells, anti-Ki67-Fitc (ThermoFisher 11-5698) for proliferating GC cells, and anti-CD3 (Abcam 5690, Cambridge, UK) for Tfh, which were counted within Ki67^+^ GC (activated Tfh) and the MZ (resting Tfh) regions as demonstrated in **Figure 4A**. Secondary antibodies used were donkey anti-rabbit-biotin (Dianova, 711-065-152, Hamburg, Germany) and AlexaFluor594 Tyramide SuperBoost Kit, strepavidin (ThermoFisher B40935) for anti-IL-21, goat anti-mouse-IgM-Cy3 (Dianova 115-166-075) for anti-CD20 and donkey anti-rabbit-Cy5 (Dianova 711-175-152) for anti-CD3. Digital pictures were assessed using Histoquest**^®^** software.

### Real-Time Quantitative PCR (qPCR)

QPCR was performed as previously described (28). Briefly, total RNA was extracted from homogenized frozen tissue using Nucleo Spin RNA Plus Kit^®^ (Macharey Nagel 740984.250, Düren, Germany). DNase was added to remove genomic DNA. Total RNA was reverse transcribed into cDNA. RT-PCR was performed on ViiA7 detection system (Applied Biosystems, Darmstadt, Germany) using QuantiTect SYBR Green PCR Kit (Qiagen, Hilden, Germany). All water controls were negative for target and housekeeper. **Table S1 (Supplementary Material)** contains primer sequences. Target gene copy numbers were normalized to house-keeper hypoxanthine-guanine phosphoribosyl-transferase (HPRT) and expressed by calculating delta CT values.

### Interleukin-21 Immunoassay

Serum IL-21 concentrations were measured using a commercial anti-rat IL-21 chemiluminescent immunoassay (Cloud clone SCB688Ra, Houston, USA) according to the manufacturer’s instructions.

### Statistical Analysis

Data is shown as individual values and mean or mean ± SEM. Statistical analysis was performed using GraphPad Prism (Version 8.0, San Diego). Mann-Whitney U-test was used and p ≤ 0.05 was considered to be statistically significant.

## Results

### Development of Allograft Rejection, Fibrosis and DSA

We used low dose CsA treatment (loCNI) to prevent fatal allograft failure due to acute rejection, but allow DSA formation. High dose CsA treatment (hiCNI) was used to suppress alloimmune functions in a non-rejecting group. An overview of experimental treatments is shown in **Figure 1A**. Mean CsA trough levels in CsA treated rats were 93 ± 34 ng/ml with 5mg/kg/d and 733 ± 135 ng/ml with 10 mg/kg/d. HiCNI treatment prevented cellular rejection (TCMR) and ABMR (**Table 2**). In contrast, 6 of 8 loCNI rats developed borderline rejection, one showed Banff IB TCMR and one showed no rejection. A portion of loCNI rats (3/8) also showed signs of active and chronic ABMR, including peritubular capillaritis, transplant glomerulitis, chronic transplant glomerulopathy and acute tubular necrosis, scored using Banff lesion criteria (**Table 2**, **Figure 1B**). C4d staining was negative in all sections (not shown).

**Table 2:** Histopathological evaluation of allograft sections in analogy to Banff classification 2017.

Group	no rejection	TCMR		ABMR	ABMR lesion scores				
		Borderline	Banff IB		ptc	g	cg	MVI^#^	ATN
hiCNI	6/6	–	–		0 ± 0	0 ± 0*	0 ± 0*	0 ± 0*	0.13 ± 0.1°
loCNI	1/8	6/8	1/8	3/8	0.5 ± 0.26	0.25 ± 0.16	0.25 ± 0.25	0.75 ± 0.4	0.63 ± 0.3

TCMR, t cell mediated rejection; ABMR, antibody mediated rejection; ptc, peritubular capillaritis; g, glomerulitis; cg, glomerulopathy; MVI, microvascular inflammation; ATN, acute tubular necrosis; n.a., not assessed.

*1 of 6 Ktx sections from this group did not contain glomeruli.

°1 section with ATN contained parenchymal necrosis, interstitial edema, subcapsular hemorrhage and signs of vascular congestion.

^#^MVI (microvascular inflammation) score = sum of ptc and g scores.

In addition, we observed a significant increase of fibrosis in kidneys after loCNI treatment compared to hiCNI treatment (1.9 ± 0.1% vs. 1.4 ± 0.1%, p=0.01) and controls (1.9 ± 0.1% vs. 0.6 ± 0.2%, p=0.01). HiCNI kidneys also showed more fibrosis than controls (p=0.048) (**Figure 1C**).

Furthermore, MFI of both IgM and IgG DSA were significantly elevated in loCNI compared to hiCNI (IgM 23.7 ± 2.1 vs. 11.9 ± 0.8, p=0.0027; IgG 59.0 ± 11.6 vs. 8.3 ± 0.5, p=0.0007) and controls (**Figures 1D, E**). The analysis of DSA IgG subclasses showed that IgG2b DSA were most prominent, followed by IgG1 (**Figures 1D, E**). In hiCNI, DSA MFI did not exceed unspecific background levels of the control group in any DSA analysis (**Figures 1D, E**). A limitation in our methodology is that only DSA against MHC class I were measured; therefore conclusions about the development and effect of MHC class II DSA cannot be drawn in our study.

### T Cell, but Not B Cell, Frequency Is Reduced in hiCNI

**Figure 2:**
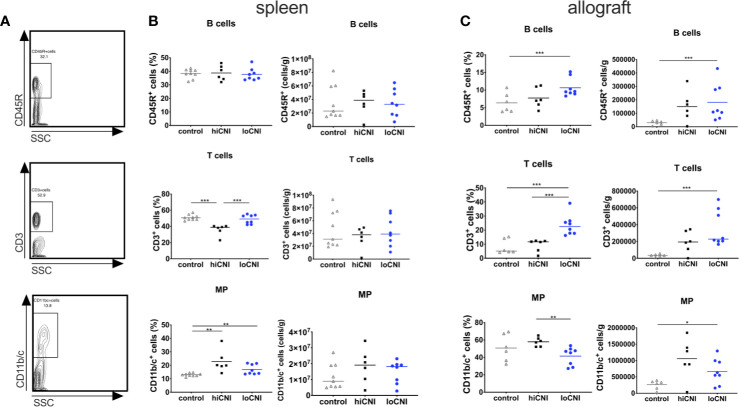
Frequency and absolute numbers of leukocyte subsets in spleens and allografts. Representative flow cytometry data **(A)**, frequency and absolute numbers of B cells (CD45R^+^), T cells (CD3^+^) and mononuclear phagocytes (MP, CD11b/c^+^) in spleen **(B)** and allografts **(C)** measured by flow cytometry and shown as percentages of leukocytes or absolute numbers per weight. Means and individual data points are shown from controls (LEW spleen n=9, BN kidneys n=6), hiCNI (n=6) and loCNI (n=8); statistical significance between groups is shown as *p≤ 0.05, **p<0.01 and ***p<0.001.

Since DSA formation was strongly inhibited by hiCNI treatment, we first examined how B cell, T cell and mononuclear phagocyte (MP) populations in the SLO were affected by experimental treatments. The frequency of B cells was not altered by CNI treatment and transplantation (**Figures 2A, B**); however, the frequency of splenic T cells was significantly reduced in hiCNI compared to loCNI (36.0% ± 2.7% vs. 48.8% ± 2.0%, p=0.0007) and controls (36.0% ± 2.7% vs. 51% ± 1.1%, p=0.0004) (**Figures 2A, B**). Both hiCNI and loCNI showed an increased proportion of MP compared to controls (loCNI vs. control: 16.9% ± 1.3% vs. 13.0% ± 0.4%, p=0.006; hiCNI vs. control: 22.9% ± 3.4% vs. 13.0% ± 0.4%, p=0.0016). There was no significant difference in the absolute numbers of splenic B or T cells or MP (**Figure 2B**). Within allografts, the frequencies and absolute numbers of both B and T cells were increased in loCNI compared to controls (B cells p=0.029 and T cells p=0.0007) (**Figure 2C**). T cell frequency was also increased in loCNI compared to hiCNI (p=0.0007), while MP frequency was decreased in loCNI compared to hiCNI (p=0.003) (**Figure 2C**).

### Germinal Centers Correlate With DSA

**Figure 3:**
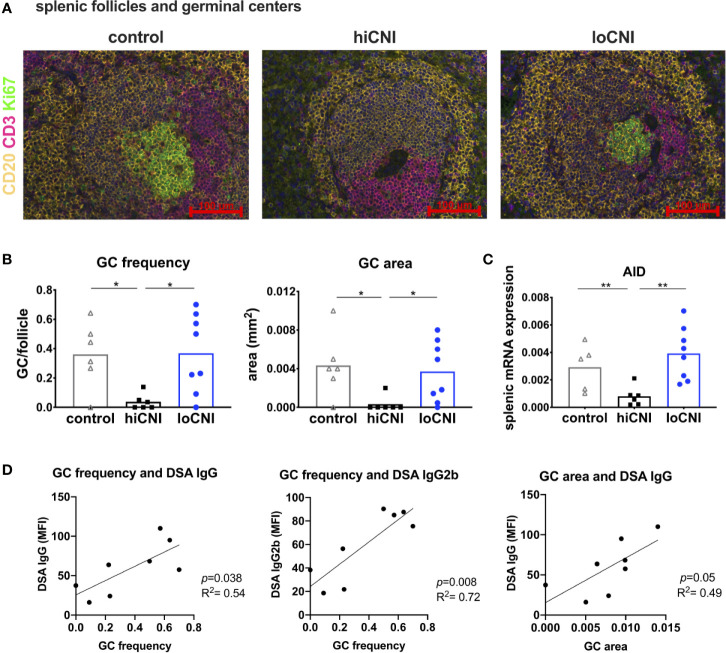
Germinal center formation. **(A)** immunofluorescence staining of splenic follicles with T cell zone (CD3^+^, red), B cell follicle (CD20^+^, yellow) and GC (Ki67^+^, green) and **(B)** average frequency and area of GC per follicle from controls (n=6), hiCNI (n=6) and loCNI (n=8). **(C)** splenic AID mRNA expression measured by qPCR in controls (n=5), hiCNI (n=6) and loCNI (n=8), normalized to house-keeping gene HPRT and expressed as delta CT (AU). Means and individual data points are shown; statistical significance between groups is shown as *p≤ 0.05 and **p<0.01. **(D)** Correlation of GC frequency or area with IgG and IgG2b DSA MFI within the loCNI group.

Since B cell frequency was not affected, we examined B cell activation in GC. We analyzed splenic follicles by immunofluorescence microscopy, and determined the frequency and size of GC (**Figures 3A, B**). The follicular area and mantle zone (MZ) area were unchanged in transplanted rats compared to untreated controls, regardless of CNI treatment (**Supplementary Material Figure S1A, B**); however, GC frequency and area were almost completely diminished in hiCNI treated rats compared to loCNI rats (GC frequency: 0.04 ± 0.02 vs. 0.37 ± 0.09, p=0.013; GC size: 0.0003 ± 0.0008 mm^2^ vs. 0.0037 ± 0.001 mm^2^, p=0.018) and controls (GC frequency: 0.04 ± 0.02 vs. 0.36 ± 0.09, p=0.028; GC size: 0.0003 ± 0.0008 mm^2^ vs. 0.004 ± 0.001 mm^2^, p=0.015) (**Figure 3B**). In line with this, splenic mRNA expression of AID (activation-induced cytidine deaminase), an essential enzyme for germinal center B cells, was also diminished in hiCNI compared to loCNI (0.0008 ± 0.0003 vs. 0.004 ± 0.0007, p=0.003) and controls (0.0008 ± 0.0003 vs. 0.003 ± 0.0008, p=0.017) (**Figure 3C**). We were interested in the relationship between DSA formation and GC frequency and expansion. We found that within the loCNI group, GC frequency correlated with both IgG and IgG2b DSA MFI, and that GC area correlated with IgG DSA MFI (p=0.05, R^2 ^= 0.49) (**Figure 3D**).

### Activated Tfh Act as Drivers of GC Formation

**Figure 4:**
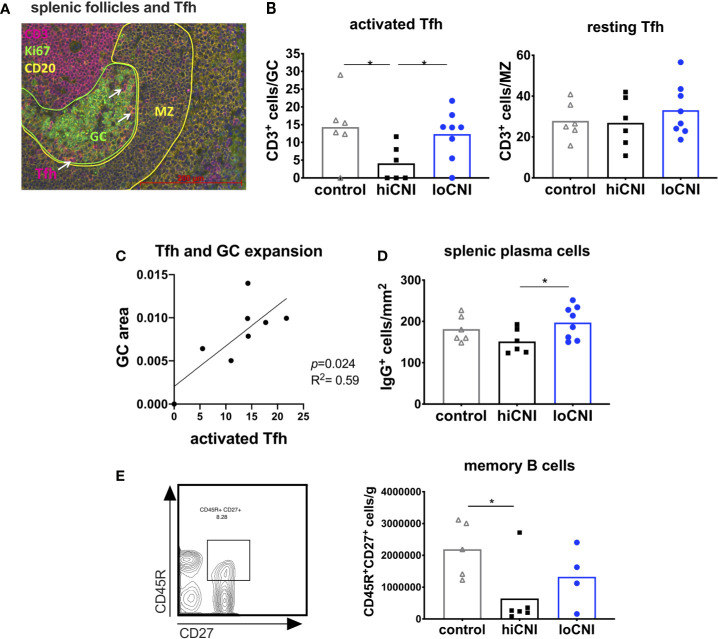
Activated T follicular helper cells drive GC formation. **(A)** immunofluorescence staining of a splenic follicle with T cell zone (CD3^+^, red), GC (Ki67^+^, circled green), B cell follicle (CD20^+^, yellow) with mantle zone (MZ, circled yellow) and Tfh (white arrows). **(B)** average number of activated Tfh (CD3^+^ cells in Ki67^+^ GC area) and resting Tfh (CD3^+^ cells in CD20^+^ MZ area) in controls (n=6), hiCNI (n=6) and loCNI (n=8). **(C)** Correlation of the number of activated Tfh and GC expansion within the loCNI group. **(D)** plasma cells measured as IgG^+^ cells in spleen sections from controls (n=6), hiCNI (n=6) and loCNI (n=8), and **(E)** CD45R^+^CD27^+^ memory B cells measured by flow cytometry in controls (n=5), hiCNI (n=6) and loCNI (n=4). Means and individual data points are shown; statistical significance between groups is shown as *p≤ 0.05.

Since GC formation was inhibited by hiCNI treatment, we investigated the signals necessary for B cell activation. *In vivo* tracking studies by Suan et al. demonstrated that Tfh within the GC have an activated phenotype, while Tfh in the mantle zone (MZ) have a resting memory Tfh phenotype (33). We found that the number of activated GC Tfh was substantially reduced in the hiCNI group compared to loCNI (4.1 ± 2.0 vs. 12.3 ± 2.4 CD3^+^ cells/GC, p=0.031) and controls (4.1 ± 2.0 vs. 14.3 ± 3.8 CD3^+^ cells/GC, p=0.028) (**Figure 4B**), while the number of resting Tfh localized within the MZ was not affected by treatments (**Figure 4B**). Since Tfh activate B cell proliferation, we were interested in the relationship between Tfh and GC expansion. We found that in loCNI, the number of activated Tfh determined the size of GC (**Figure 4C**).

Plasma cells and memory B cells are the products of the GC reaction. As a consequence of inhibited GC formation in hiCNI, the number of splenic plasma cells was also lower in hiCNI compared to loCNI (160 ± 15 cells/mm^2^ vs. 169 ± 11 cells/mm^2^, p=0.04) (**Figure 4D**). The number of splenic memory B cells was non-significantly reduced in hiCNI compared to loCNI (6.4x10^5^ ± 4.2x10^5^ vs. 13.3x10^5^ ± 4.7x10^5^, p=0.47) (**Figure 4E**).

### HiCNI Inhibits Markers of Tfh Differentiation and Function

**Figure 5:**
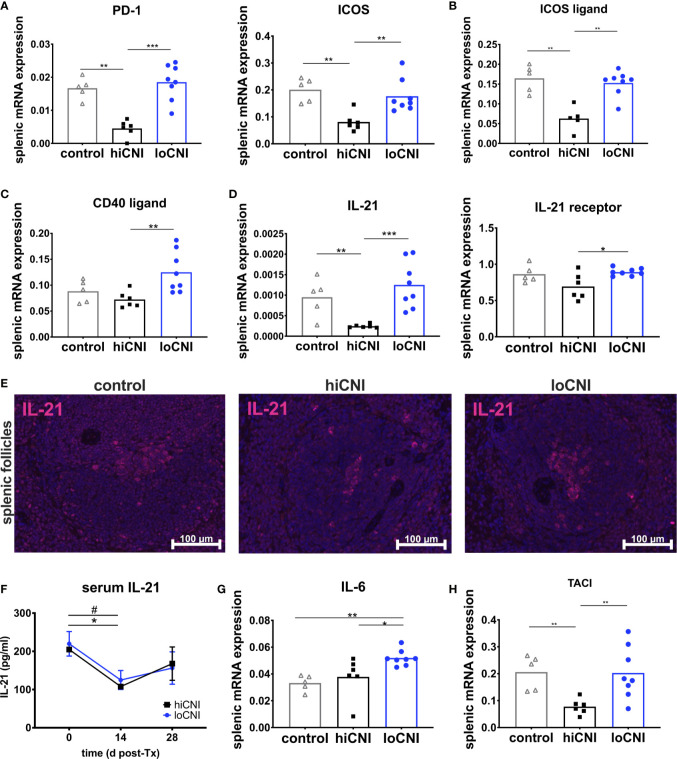
Markers associated with activation and function of T follicular helper cells. **(A)** splenic mRNA expression of PD-1 and ICOS, **(B)** ICOS ligand **(C)** CD40 ligand and **(D)** IL-21 and IL-21 receptor. From controls (n=5), hiCNI (n=6) and loCNI (n=8), **(E)** representative immunofluorescent staining of IL-21 within splenic follicles. **(F)** Serum IL-21 concentrations of hiCNI and loCNI at d0 (hiCNI n=3, loCNI n=3), d14 (hiCNI n=2, loCNI n=8) and d28 (hiCNI n=6, loCNI n=8) post-Ktx. Splenic mRNA expression of IL-6 **(G)** and TACI **(H)** from controls (n=5), hiCNI (n=6) and loCNI (n=8). Expression of mRNA was normalized to house-keeping gene HPRT and expressed as delta CT (AU). Data is shown as mean and individual data points; statistical significance is denoted as *p≤ 0.05, **p<0.01 and ***p<0.001. In **(F)**, ^#^p≤0.05 for loCNI, *p≤0.05 for hiCNI.

Tfh arise after activation of naïve T cells, which was inhibited in hiCNI, as demonstrated by a substantially reduced expression of markers associated with T cell activation, PD-1 (programmed cell death protein 1) and ICOS (inducible T cell costimulator) in hiCNI compared to loCNI (PD-1: 0.005 ± 0.001 vs. 0.018 ± 0.002, p=0.0007; ICOS: 0.08 ± 0.014 vs. 0.18 ± 0.02, p=0.005) and controls (PD-1: 0.005 ± 0.001 vs. 0.017 ± 0.001, p=0.004; ICOS: 0.08 ± 0.001 vs. 0.20 ± 0.02, p=0.004) (**Figure 5A**). Correspondingly, mRNA expression of the T cell costimulatory receptor CD28 and Th lineage transcription factors GATA3 (GATA binding protein 3) and Foxp3 (forkhead box protein P3) was also significantly lower in hiCNI compared to loCNI (**Supplementary Material Figure S2A**).

Signals from B cells, such as ICOS ligand, are required to promote Tfh differentiation and maintenance (34, 35). We found that mRNA expression of ICOS ligand was significantly diminished in hiCNI compared to loCNI (0.063 ± 0.014 vs. 0.15 ± 0.01, p=0.003) and compared to controls (0.063 ± 0.014 vs. 0.16 ± 0.015, p=0.008) (**Figure 5B**).

Next, we were interested in the expression of molecules associated with Tfh effector function. Although CD40 ligand mRNA expression was significantly lower in hiCNI than in loCNI (0.07 ± 0.006 vs. 0.13 ± 0.014, p=0.005), it was not suppressed by hiCNI treatment in comparison to controls (**Figure 5C**). Splenic IL-21 mRNA expression, on the other hand, was potently inhibited by hiCNI treatment compared to loCNI (0.0002 ± 0.000002 vs. 0.0013 ± 0.0002, p=0.0007) and controls (0.0002 ± 0.000002 vs. 0.001 ± 0.0002, p=0.009). Interestingly, the mRNA expression of the IL-21 receptor was also lower in hiCNI treatment compared to loCNI (0.69 ± 0.07 vs. 0.89 ± 0.02, p=0.029) (**Figure 5D**). Using immunofluorescence microscopy, we showed that follicular IL-21 expression was significantly less prominent in hiCNI compared to loCNI or controls (IL-21^+^ area in hiCNI vs. loCNI: 0.19 ± 0.18 mm^2^ vs. 0.9 ± 0.34 mm^2^, p=0.04) (hiCNI vs. control: 0.19 ± 0.18 mm^2^ vs. 1.5 ± 0.31 mm^2^, p=0.013) (**Figure 5E**). In serum, there was no significant difference in IL-21 concentrations between hiCNI vs. loCNI, but a decrease in IL-21 levels was noted in both CNI groups after transplantation (d14), possibly mediated by CsA treatment (**Figure 5F**). Surprisingly, splenic mRNA expression of cytokines associated with T helper cell subsets, IL-2, IFN-γ (interferon-γ), IL-4 and TGF-β (transforming growth factor-β), was not reduced by hiCNI treatment (**Supplementary Material Figure S2**).

### Other Axes Involved in T:B Crosstalk and Humoral Immune Activation

We were interested in other important axes that activate humoral immunity. Interleukin(IL)-6 is secreted by B cells and supports Tfh maintenance. We found that IL-6 was elevated in loCNI compared to hiCNI (0.052 ± 0.002 vs. 0.038 ± 0.006, p=0.02) and controls (0.052 ± 0.002 vs. 0.033 ± 0.003, p=0.002), but its expression was not inhibited by hiCNI compared to controls (**Figure 5G**). BAFF (B cell activating factor), its homolog APRIL (a proliferation inducing ligand) are both drivers of humoral immunity. We measured expression of BAFF, APRIL and their receptors BAFF-R (BAFF receptor), BCMA (B cell maturation antigen) and TACI (transmembrane activator and calcium modulator and cyclophilin ligand interactor), and found that expression of TACI, but not BAFF, APRIL, BAFF-R or BCMA, was significantly reduced in hiCNI compared to loCNI (p=0.005) or control (p=0.004) (**Figure 5H**).

## Discussion

We used a unique model of ABMR based on *de novo* sensitization to study the mechanisms of humoral alloimmune activation *in vivo*. Using this model closely resembling human Ktx, we investigated the relationship between GC, Tfh and DSA formation in SLO and the effects of CNI dose variation on key effector cells and molecules. We found that DSA formation and ABMR development were prevented in rats treated with hiCNI, while loCNI treatment did not hinder DSA formation and only partially prevented ABMR. GC formation was inhibited in hiCNI, but permitted in loCNI. As a result, plasma cell output from GC was reduced in hiCNI compared to loCNI. Fittingly, the number of activated Tfh was significantly reduced in hiCNI. In addition, we found that expression of Tfh maturation and maintenance factors ICOS ligand and IL-21 was also lower in hiCNI compared to loCNI. Follicular expression of the Tfh effector cytokine IL-21 was substantially inhibited in hiCNI. In summary, our results highlight the role of Tfh and GC in the development of *de novo* DSA and show that ABMR is prevented when Tfh:B cell crosstalk is disrupted.

In our model, low dose CNI-based immunosuppression was administered to prevent fatal allograft rejection. All rats with loCNI treatment, but none with hiCNI treatment, developed DSA. In addition, loCNI led to borderline cellular rejection in most rats and acute and/or chronic ABMR in a portion of rats. Kidney fibrosis was increased by transplantation and was potentiated by rejection in rats with loCNI. Therefore, our model displayed many features of chronic rejection seen in Ktx patients.

The activation of Tfh and B cells is intricately linked and depends on T:B interactions within SLO. These interactions require the specific movements and spatial arrangements of T and B cells within lymphoid follicles. We chose to examine spleens as representative SLO to visualize T:B interactions, based on previous data showing that allo-antigens from vascularized allografts can be detected in higher amounts in spleen than in draining lymph nodes (36). We found that B cell proliferation in GC was virtually eliminated by hiCNI, but not loCNI treatment, which did not prevent GC formation. Moreover, we showed that DSA MFI correlated with the frequency and size of GC. We also observed GC formation in the untreated controls, but this is common in healthy rats housed in conventional research environments (37). Furthermore, we found a lower number of splenic plasma cells in hiCNI compared to loCNI, which we interpreted as a result of reduced output from GC. Our results point out the dependency of DSA generation on GC formation and highlight the impact of CNI dose variation on GC formation.

Tfh are non-redundant drivers of GC formation (38, 39). Since DSA did not form in the absence of GC, our data supports a T-dependent mechanism of alloantibody formation *via* the GC. In line with our observations, a recent report showed a requirement for Tfh for humoral alloresponses in a murine heart transplant model (40). In our study, activated Tfh were virtually absent in hiCNI treated rats. Furthermore, we showed that the number of activated Tfh determined the expansion of GC. While the number of activated GC Tfh was substantially diminished in hiCNI, we found that resting MZ Tfh cell numbers were not affected by immunosuppressive treatment. Overall, our data underline the important role of Tfh in the formation of DSA.

Tfh follow a distinctive differentiation pathway, requiring a number of signals, as reviewed by Wali (41). Development of Tfh is initiated by antigen-presentation and cytokine priming of naïve T helper (Th) cells by dendritic cells. In our model, the diminished number of activated Tfh in hiCNI may have resulted from inhibition of naïve T cell activation or a deficit in Tfh maturation or maintenance signals. We found reduced expression of markers associated with T cell activation, PD-1 and ICOS, in hiCNI indicating inhibition of T cell activation. In addition, expression of other Th cell subset lineage markers, such as GATA3 and Foxp3, was also significantly reduced in hiCNI, suggesting a common mechanism of inhibition of naïve T cell activation by CNI, as previously described (42). Overall, our results suggest that CNI treatment has an inhibitory effect on *de novo* development of Tfh. Indeed, suppression of Tfh formation by CNI has been shown *in vitro* (43, 44). Our results show that Tfh lineage development was highly susceptible to high dose CNI treatment.

However, hiCNI treatment also inhibited the expression of Tfh maturation and maintenance signals. Pre-Tfh upregulate the B-cell chemokine receptor CXCR5 (45), which guides them to the T:B border in lymphoid follicles. Here, pre-Tfh receive essential signals from B cells enabling full maturation of Tfh (46). While ICOS is expressed during T cell activation, ICOS ligand is induced in activated B cells and is required for Tfh maturation into a fully functional state (46, 47). Our results showed a significant inhibition of ICOS ligand expression. Previous reports showed that CNI can inhibit naïve B cell activation directly *in vitro* (43), making a B-cell mediated indirect effect of hiCNI on Tfh differentiation a distinct possibility. We show that Tfh:B cell crosstalk *via* the ICOS – ICOS ligand axis is bilaterally disrupted by hiCNI treatment.

Tfh provide essential activation signals to cognate B cells *via* costimulatory molecules and the secretion of cytokines. CD40 ligand and IL-21 are the key Tfh effector molecules required for B cell activation. Interestingly, while IL-21 expression was potently inhibited by hiCNI treatment, CD40 ligand expression was not. IL-21 not only functions as a key B cell activating cytokine and driver of GC formation, it also stimulates Tfh maturation in an autocrine manner (48). We show for the first time a substantial inhibition of IL-21 expression in SLO by hiCNI treatment *in vivo*, while IL-2 and IFN-γ expression was not significantly affected. Fittingly, the expression of the IL-21 receptor was also reduced in hiCNI, reflecting a lack of autocrine Tfh stimulation and abrogated B cell activation. Since Tfh generation depends on IL-21, the shortage of IL-21 produced in hiCNI may have stifled Tfh generation in our model.

Another important factor for Tfh maintenance is IL-6. IL-6 is secreted by B cells and plasmablasts and acts as an amplifier of Tfh-dependent B cell activation in a positive feedback loop (35). We found that IL-6 expression in SLO was elevated in loCNI, but was not inhibited by hiCNI below control levels. Our findings suggest that prolonged alloimmune activation and inflammation leads to IL-6 elevation, which can pathologically amplify the humoral response. Tocilizumab, a monoclonal anti-IL-6 antibody, has recently shown promising initial results for the treatment of chronic ABMR in Ktx patients (49). Our results demonstrate that IL-6, an important amplifier of humoral responses, is not targeted by CNI treatment.

Finally, we were interested in the involvement of the BAFF/APRIL axis, which has been linked to pre-transplant sensitization, DSA occurrence and ABMR in Ktx patients and experimental Ktx (30, 50–53). We found no change in BAFF, APRIL, BAFF-R or BCMA expression in SLO, but the expression of the BAFF/APRIL receptor TACI was significantly reduced in hiCNI. Our results demonstrate that the BAFF/APRIL axis is only partially affected by CNI treatment *via* reduced TACI expression.

In summary, our study highlights the important role of Tfh:B cell crosstalk and the effects of CNI dose variation on humoral alloimmune activation *in vivo*. Overall, our data supports targeting Tfh or its effector molecules to prevent ABMR and improve long-term outcomes in Ktx patients.

## Data Availability Statement

The raw data supporting the conclusions of this article will be made available by the authors, without undue reservation.

## Ethics Statement

The animal study was reviewed and approved by Regierung von Unterfranken.

## Author Contributions

LS designed experiments, performed data analysis and interpretation and wrote the manuscript. HP performed rat operations and treatments. AS contributed to data analysis and reviewed the manuscript. KA evaluated histopathology and reviewed the manuscript. BB contributed to data interpretation and reviewed the manuscript. TB contributed to experimental design and data interpretation and reviewed the manuscript. All authors contributed to the article and approved the submitted version.

## Funding

This work was funded by the Deutsche Forschungsgemeinschaft (DFG, German Research Foundation), project number 387509280, SFB 1350 Project B6 to BT and BB.

## Conflict of Interest

The authors declare that the research was conducted in the absence of any commercial or financial relationships that could be construed as a potential conflict of interest.
