# Supplementary material for: Disruption of Tfh:B Cell Interactions Prevents Antibody-Mediated Rejection in a Kidney Transplant Model in Rats: Impact of Calcineurin Inhibitor Dose
Source: Front Immunol. 2021 May 31;12:657894. doi: 10.3389/fimmu.2021.657894 (PMC8201497; doi:10.3389/fimmu.2021.657894)
Supplement: Supplementary file 1 [file DataSheet_1.docx]

Supplementary Material

**Supplementary Figure 1.**

B cell follicles and Tfh. Using immunofluorescence staining of a splenic follicle with T cell zone (CD3^+^, red), GC (Ki67^+^, circled green), B cell follicle (CD20^+^, yellow) with mantle zone (MZ, circled yellow), and Tfh (white arrows) as shown in fig. 4a, (A) shows average total follicular area (GC and MZ zone) and average MZ area, (B) average number of Tfh in GC and MZ combined. Means and individual data points are shown; statistical significance between groups is shown as *p≤0.05, **p<0.01 and ***p<0.001.

**Supplementary Figure 2.**

T helper cell subset transcription factors and cytokines. (A) splenic mRNA expression of CD28, (B) transcription factors T-bet (Txb21), Gata-3 and foxp3, and (C) cytokines IL-2, IFN-γ, IL-4 and TGF-β. Expression of mRNA was normalized to house-keeping gene HPRT and expressed as delta CT (AU). Data is shown as mean and individual data points; statistical significance is denoted as *p≤0.05, **p<0.01 and ***p<0.001.

**Table S1: PCR Primer Sequences:**

rHPRT forw (5'-CTTTGGTCAAGCAGTACA GCC-3')

rHPRT rev (5'-TCCGCTGATGACACAAACATGA-3')

rAID forw (5'-CTT GAA GCA AGC TCC CTT TG-3')

rAID rev (5'-GCG GAC ATT TTT GAA GTG GT-3')

rAPRIL forw (29) (5'- ATC CTG ACC GTG CCT ACA AC -3')

rAPRIL rev (30) (5'- TCA CAA ACC CCA GGA ATG TT -3')

rBAFF_for: (5'- GAC CGG AGG AAA CAG AAC AA -3')

rBAFF_rev: (5'- TGC AAT CAG CTG CAG ACA GT -3')

rBAFF-R forw2 (73) (5'-GTG GGT CTG GTG AGT CTG GT-3')

rBAFF-R rev2 (74) (5'-CAT TTT CCA GGG ACT CTT GC-3')

rBCMA forw (5'-CGT CTG TTT GGC ACT TTT CA-3')

rBCMA rev (5'-CAC CAG CCC TGC TCT TAG TC-3')

rCD28_forw (5‘-AGTCCTGCTGTGTTATGGCT-3’)

rCD28_rev (5‘-GTACGCTGCAAAGTCTCTCG-3’)

rCD40Lig_forw2 (5‘-AGA TGA TTG GGT CGG TGC TT-3’)

rCD40Lig_rev2 (5‘-CCC TTC TCC TTT GTT GCA CC-3’)

Gata-3_forw (5‘-CGG AAG AGG TGG ACG TAC TT-3’)

Gata-3_rev (5‘-ATG GAC GTC TTG GAG AAG GG-3’)

rFoxp3_for (5’-CTA TGC CAC CCT CAT CCG AT-3’)

rFoxp3_rev (5’-ACA CTG CTC CCT TCT CAC TC-3’)

rICOS_forw2 (5‘-AAT CCC AGC TTT GTT GCC AG-3’)

rICOS_rev2 (5‘-TCG TGC ACA CTG GAT CTG TA-3’)

rICOSLig_forw (5'-TGG ATC AAC AGG ACG GAC AA-3')

rICOSLig_rev (5'-GGA TTT CCT GTG GCC TCT CT-3')

rIFN-g_forw2 (5’-GTG TCA TCG AAT CGC ACC TG-3’)

rIFN-g_rev2 (5’-CTT TGT GCT GGA TCT GTG GG-3’)

rIL-2 forw1 (5’-AAG GAA ACA CAG CAG CAC CT-3’)

rIL-2 forw1 (5’-GTG AGC ATC ATG GGG AGT TT-3’)

rIL-4 forw1 (5’-CGTGATGTACCTCCGTGCT-3’)

rIL-4 forw1 (5’-AGTGTTGTGAGCGTGGACT-3’)

rIL-6_forw (5'-AGC CAG AGT CAT TCA GAG CA-3')

rIL-6_rev (5'-AGA GCA TTG GAA GTT GGG GT-3')

rIL-21_BT forw2 (5’-CGA AGC TTT TGC CTG TTT TC-3’)

rIL-21_BT rev2 (5’-CAA ATC ACA GGA AGG GCA TT-3’)

rIL-21-rec_forw (5’-GAGAGTTTCTTCCAGCCCCT-3’)

rIL-21-rec_rev (5’-GACCAGGTCCAGACATTCCA-3’)

rPD-1_forw2 (5’-CCAGAAGGCCAGTTTCAAGG-3’)

rPD-1_rev2 (5’-CTGAGCCCCTACGTCCTATG-3’)

rTGF-b_forw2 (5’-CAT GCC AAC TTC TGT CTG GG-3’)

rTGF-b_rev2 (5’-CGG GTT GTG TTG GTT GTA G-3’)

Txb-21(T-bet)_for (5‘-GTC CTG CAG TCC CTC CAT AA-3’)

Txb-21(T-bet)_rev (5‘-GGA CTC AAA GTT CTC CCG GA-3’)

rTACI forw1 (5'- GGC CGG ATA ACT TAG GAA GG -3')

rTACI rev1 (5'- TGG GAA GTG GCT CTC CTC TA-3')
